# ProBDNF/p75NTR/sortilin pathway is activated in peripheral blood of patients with alcohol dependence

**DOI:** 10.1038/s41398-017-0015-4

**Published:** 2018-03-09

**Authors:** Li Zhou, Jing Xiong, Chun-Sheng Ruan, Ye Ruan, Dennis Liu, Jian-Jun Bao, Xin-Fu Zhou

**Affiliations:** 1 The Mental Hospital of Yunnan Province, Kunming, Yunnan Province China; 2 Institute of Molecular and Clinical Medicine, Kunming Medical University, Kunming, Yunnan Province China; 3 Department of Neurology, the Second Affiliated Hospital, Kunming Medical University, Kunming, Yunnan Province China; 4 School of Pharmacy and Medical Sciences, Division of Health Sciences, University of South Australia, Adelaide, SA Australia; 5 Discipline of Psychiatry, School of Medicine, University of Adelaide, Adelaide, SA Australia; 6 Mental Health Service, Northern Adelaide Local Health Network, Adelaide, SA Australia

## Abstract

Alcohol dependence is a worldwide problem with a great social and economic burden in many countries. A number of studies have suggested that BDNF (mature BDNF) and its precursor (proBDNF) play important roles in the alcohol dependence. However, what roles of the mBDNF/proBDNF pathways play during the pathological process of alcohol dependence are not clearly understood. In our clinical study, peripheral blood was sampled from 30 male patients with alcohol dependence and 50 healthy males (as control). The protein levels of proBDNF, p75NTR, sortilin, mBDNF, TrkB and mRNA levels of *BDNF*, *p75NTR*, *sortilin*, and *TrkB* were detected in the peripheral blood in our study. We found that the protein levels of proBDNF and p75NTR were increased, but not the sortilin protein level; while the TrkB protein level was decreased in the alcohol dependence patients compared with healthy controls. Moreover, the mRNA levels of *p75NTR* and *sortilin* from the lymphocytes were slightly increased; while *BDNF* and *TrkB* were significantly decreased. The ELISA results of mBDNF and TrkB were declined in the alcohol dependence group. The levels of mBDNF and TrkB were negatively correlated with the average amount of daily ethanol consumption, and the levels of proBDNF, p75NTR and sortilin were positively correlated with the average amount of ethanol consumption per day. The ratio of proBDNF to mBDNF was altered in alcohol dependence patients. The balance between the proBDNF/p75NTR and mBDNF/TrkB signalling pathways appeared dysregulated in alcohol dependence. Our results suggested that both pathways may participate in the complex processes of alcohol dependence.

## Introduction

Brain-derived neurotrophic factor (BDNF) is under a pivotal role in the psychiatric disorders, including alcohol dependence^1–3^. Alcohol dependence or alcohol abuse is a worldwide problem with a great social and economic burden in many countries. Gabbard has stated in his book that “The most common substance of abuse/dependence in patients presenting for treatment is alcohol.”^4^ The aetiology of alcohol dependence is not clear, but it is known that both genetic causes of predisposition and environmental factors play critical roles in it^5^. Nonetheless, how the genetic and environmental factors interact to promote the pathogenesis of alcohol dependence requires further investigations.

Human alcoholics, in both men and women show a significant volume loss (shrinkage) in cortical and subcortical brain structures, which include both grey and white matters. Ethanol can also cause neuronal cell death, impair differentiation, reduce neuronal numbers and weaken neuronal plasticity. BDNF can significantly reverse the ethanol-induced neuronal toxicity^6–8^. BDNF is synthesised as a precursor molecule (proBDNF), which is enzymatically processed to mature BDNF (mBDNF) and BDNF predomain. ProBDNF and mBDNF play different, even opposite roles in neuronal survival, differentiation and plasticity^9^. Many studies show that mBDNF and its high-affinity receptor, tyrosine kinase receptor (TrkB), play pivotal roles in mediating neuronal survival and growth. Its precursor (proBDNF) can induce neuronal apoptosis via binding with p75 neurotrophin receptor (p75NTR) and sortilin^10–12^. Little is known about their levels in the diseased conditions. Chronic ethanol exposure can also remodel the *BDNF* gene at the chromatin level, contribute to the development and maintenance of addiction^13–15^. The BDNF and TrkB levels decreased in alcohol-dependent patients, increased after abstinence. Furthermore, the p75NTR level was the opposite of those of BDNF and TrkB during alcohol dependence and abstinence^16^. Moreover, excessive alcohol intake would lead to neuroadaptation by destructing BDNF/TrkB signalling pathway. When given p75NTR modulator, the excessive alcohol intake would significantly reduce in rats^17^. Since proBDNF and mBDNF have opposite functions, expression levels of proBDNF and mBDNF in alcohol dependence also should be distinguished. We postulate that chronic ethanol exposure can alter the metabolism of BDNF, leading to the imbalance of proBDNF vs. mBDNF and the changes of signalling pathways of proBDNF/p75NTR/sortilin and mBDNF/TrkB. In the present study, we have tested this hypothesis in male patients with alcohol dependence.

## Materials and methods

### Subjects

The study was based on clinical samples, which was approved by the Ethics Committee of the Mental Hospital of Yunnan Province, and all the experimental procedures were conducted in accordance with the Helsinki Declaration of 1975, as revised in 1983. Thirty alcohol dependence male patients who went to detoxification treatment voluntarily were recruited from detoxification treatment unit in the Mental Hospital of Yunnan Province, China, from September 2010 to May 2011. Patients were enrolled to participate in the study after they had signed the informed consents for participation. The Chinese version of the Mini International Neuropsychiatric Interview (MINI) and the alcohol use disorders identification test (AUDIT) were used to screen their psychiatric and drinking conditions to confirm that they met the diagnosis of alcohol dependence according to the International Statistical Classification of Diseases and Related Health Problems 10th Revision (ICD-10) criteria. Excluded criteria were those: who were comorbidity with other current non-nicotine substance abuse or dependence, including sedatives; who used sedatives in the past one month; who had significant physical illnesses; who had other psychiatric disorders, such as schizophrenia, bipolar disorder, or major depressive disorder and suffered from severe cognitive impairment with difficulty in understanding the study content. The drinking histories such as: age at first intoxication and the average daily amount of alcohol consumption (which was amended into pure ethanol) in last one month were collected. All the alcohol dependence patients received oral Oxazepam 30–60 mg according to the severity of withdrawal symptoms to alleviate the withdrawal symptoms.

The control group (shown in Table 1) included 50 healthy male subjects without severe physical illnesses and psychiatric disorders who came to the clinic for a routine physical examination. The exclusion criteria of healthy controls were: (1) meet the diagnostic criteria of alcohol abuse/dependence in the past; (2) the average daily consumption of alcohol was more than 50 g (pure ethanol) during the last three months; (3) lifetime diagnosis of dementia, schizophrenia, schizoaffective disorder or mood disorder according to the ICD-10 by using the Chinese version of MINI and AUDIT.

**Table 1:** The demographic data of the alcohol dependence group and control group

Groups	Control (*n*=50)	Alcohol dependence (*n*=30)	*P-*value
Age (years)	25–59 43.696 ± 8.773	30–63 43.891 ± 7.522	0.172
Ethnicity	Han nationality	Han nationality	n.a.
Average lifetime drinking (years)	n.a.	16.971 ± 5.8.	n.a.
Average daily amount of ethanol consumption (gram)	0 + 50	500 + 250	< 0.001
AUDIT scores	1 + 1	23.5 + 7.25	< 0.001

There were no significant differences of the average ages between control group and alcohol dependence group. The differences of average daily amount of ethanol consumption and AUDIT scores were significant between the two groups (***P < 0.001)

*n.a.* not applicable

All the participants (Han nationality) were from the Kunming City of Yunnan Province. They were similar in age, gender and ethnicity with the alcohol dependence patients.

The mRNA levels of *BDNF*, *p75NTR*, *sortilin* and *TrkB* in lymphocytes were measured with quantitative real-time PCR; the protein levels of proBDNF, p75NTR, sortilin and TrkB in lymphocytes were measured with western blotting; and the levels of mBDNF and TrkB in the serum were measured with enzyme-linked immunosorbent assay (ELISA). Because some blood samples had not enough lymphocytes to complete both the western blotting and quantitative real-time PCR experiments, so there were 28 samples of alcohol dependence and 46 control samples in the quantitative real-time PCR experiment; there were 27 samples of alcohol dependence and 45 control samples in the lymphocytes western blotting experiment.

### Blood sampling

After having obtained the permission, the coagulant and anti-coagulant vacuum blood collection tubes with Complete Proteinase Inhibitor Cocktail (cat.#04693116001, Roche Diagnostics, Indianapolis, IN, USA), plasminogen activator inhibitor 1 (PAI-1) (cat.#A8111, Sigma-Aldrich) and the furin inhibitor I (Lot.#D00078005, Calbiochem) were used to get 2–5 ml peripheral blood on the next morning of admission.

### Isolation of serum

Human blood obtained from the antecubital vein was collected in coagulant tubes and kept on ice for 4 h, and then centrifuged at 1500×*g*, 4 °C for 10 min. The serum (top layer with light yellow colour) was carefully collected and stored at −80 °C before further experiment.

### Isolation of lymphocytes

Blood was obtained from the antecubital vein after overnight fasting in anti-coagulant tubes and kept at 4 °C. The human lymphocytes separation medium **(**cat.# P8610-200, Solarbio, China) was used to separate the lymphocytes from the blood and kept in −80 °C before total RNA and protein extraction.

### Protein isolation from lymphocytes

The detailed procedures were described in our previous paper^18^. Briefly, the samples were homogenised in RIPA buffer (containing 50 mM Tris, 150 mM NaCl, 1.0% Triton x-100, 0.5% sodium deoxycholate, 0.1% SDS) supplemented with Complete Proteinase Inhibitor Cocktail (cat.#04693116001, Roche, USA), Plasminogen Activator Inhibitor 1(PAI-1) (cat.#A8111, Sigma-Aldrich, USA) and furin inhibitor I (Lot.#D00078005, Calbiochem, USA). After centrifugation at 1500×*g* for 30 min, the supernatant was collected and the total protein was quantified using BCA Protein Assay kit (cat.#CW0014, Cwbiotech, China).

### Western blotting

We had interpreted the details in our previously paper^18^. Briefly, 50 μg lymphocyte protein was separated by running a SDS-PAGE gel, and transferred onto a polyvinylidene fluoride (PVDF) membrane (400 mA, 2 h). The membrane was blocked with 5% skimmed milk dissolved in 0.1% Tween-20/Tris-buffered saline (TBST) at room temperature for 1 h. Followed by incubation of primary antibodies (sheep anti-human proBDNF 1:200, from professor Xin-Fu Zhou’s laboratory; goat anti-human p75NTR 1:250, cat.#sc-6188, Santa Cruz, USA; rabbit anti-human sortilin 1:1000, cat.#ab16640, Abcam, USA; rabbit anti-human TrkB 1:1000, cat.#07-225, Millipore, USA; mouse anti-human beta-tubulin 1:1000, cat.#T5168, Sigma, USA) at 4 °C, overnight. After washed in TBST, the membranes were further incubated with horseradish peroxidase-conjugated anti-rabbit/mouse/sheep/goat specie-specific secondary antibodies (cat.#sc-2020, Santa Cruz, USA; cat.#CW0103, Cwbiotech, China) at room temperature for 1 h. After washed again in TBST, enhanced chemiluminescent (ECL) substrate (cat.#cw0049, Cwbiotech, China) was applied on the membrane before imaging using ChemiDoc^TM^ XRS + System (Bio-Rad, USA). For quantify the protein level in lymphocytes, beta-tubulin was blotted as loading control. The blots were analysed using ImageJ software (NIH, USA).

### Quantitative real-time PCR

The methodology of quantitative real-time PCR was described in our previous paper^18^. Briefly, the total RNA was extracted from the lymphocytes using the Trizol reagent (cat.#15596-026, Invitrogen, USA). Complementary DNA was synthesised using PrimeScript® RT reagent Kit Perfect Real Time (Code: DRR037A, TaKaRa, China) according to the manufacturer’s instruction. Gene expression levels of *BDNF*, *TrkB*, *p75NTR* and *sortilin* were detected by running quantitative real-time PCR in ABI 7300 (ABI Applied Biosystems, USA) using SYBR Green mix. The gene expression of *β-actin* was detected as internal control. The following primers were used:

*BDNF* forward: 5'-TACTTTGGTTGCATGAAGGCTGCC-3'

reverse: 5'-ACTTGACTACTGAGCATCACCCTG-3'

*TrkB* forward: 5'-AGGGCAACCCGCCCACGGAA-3'

reverse: 5'-GGATCGGTCTGGGGAAAAG-3'

*p75NTR* forward: 5'-GTGGGACAGAGTCTGGGTGT-3'

reverse: 5'-AAGGAGGGGAGGTGATAGGA-3'

*sortilin* forward: 5'-TTGATGATCTCAGAGGCTCAG-3'

reverse: 5'-TGAAGATTCTTCCTCCACGAC-3'

*β-actin* forward: 5'-CGGGAAATCGTGCGTGAC-3'

reverse: 5'-TGGAAGGTGGACAGCGAGG-3'

The primers for *BDNF*, *TrkB* and *p75NTR* were derived from Fauchais^19^ and the primers for *sortilin* were self-designed. The PCR cycling conditions were: 95 °C for 10 min, followed by 40 cycles at 95 °C for 15 s and 60 °C for 1 min. Samples were processed in technical duplicates and a melting analysis was performed in each sample at the end of PCR. The Ct value was determined for target genes (*BDNF*, *TrkB*, *p75NTR* and *sortilin*) and the endogenous control *β-actin* gene in each sample. The differences between target genes and the control Ct were determined for each sample, resulting in the ΔCt value. To determine relative differences in mRNA expression between different target genes, the 2^−ΔΔCt^ was applied.

### ELISA assay of mBDNF and TrkB

As previously described in Zhou et al.^18^ in the year of 2013 and proved by Lim et al.^20^ in the year of 2015, the ELISA steps were as following: capture antibody (protein G-purified mouse anti-mBDNF monoclonal antibody, B34D10, made in house) was diluted to 2 ug/ml in coating buffer (50 mM Carbonate) and coated to a 96-well microplate (100 ul/well). The plate was then incubated at 37 °C for 1 h. After being washed with phosphate-buffered saline (PBS) for three times, the plate was added with blocking buffer (PBS-BSA, 150 ul/well) and incubated at 37 °C for 1 h again. Afterwards, the sera and mBDNF standards (0.125–2 ug/ml) were diluted properly and added into the plate (100 ul/well). The plate was incubated at 37 °C for 1 h, and then was washed with wash buffer (four times). The detecting antibody was diluted (to 2.5 ug/ml) and added to the plate (100 ul/well). Then, the plate was incubated at 37 °C for 1 h. After being washed with wash buffer for four times, the plate was added with TMB substrate (100 ul/well). After 10–15 min, 1 N sulphuric acid was added to stop the reaction. Then, the plate was read at 450 nm in a microplate reader (Model Sunrise, TECAN, Germany). The concentration of BDNF was calculated by comparing with the standards. Serum levels of TrkB were determined by the commercial human TrkB ELISA kit (Sino Biological, Inc., Beijing, China) according to the manufacturer’s instructions.

### Statistical analyses

All statistical analyses were performed using Statistical Product and Service Solutions (SPSS) 19.0 Version. The data of average ages and average lifetime drinking was presented as mean ± SD, Student’s *t*-test was used for the comparison. The average ethanol consumption, AUDIT scores and mBDNF ELISA results was presented as median + Interquartile range (IQR), because they were not normality ones, and the Mann–Whitney *U*-test was used to analyse them. The rest results were given as mean ± standard error of mean (SEM), and Student’s *t*-test was used for the comparison. The Spearman’s correlation test was used for analysing the correlations of the average daily ethanol consumption with proBDNF, mBDNF, p75NTR, sortilin and TrkB. *P* < 0.05 (two-tailed) was considered as statistically significant.

## Results

The demographic data of the patient group and control group was shown in Table 1. There were no significant differences of the average ages between control group and alcohol dependence group (*t* = 1.373, *P = *0.172, Student’s *t*-test). The average daily amount of ethanol consumption and AUDIT scores were significantly higher in alcohol dependence group than control group (average daily amount of ethanol consumption: *z* = −8.9315.098, *P* < 0.001; AUDIT scores: *z* = −7.531, *P* < 0.001, Mann–Whitney *U*-test).

### ProBDNF/p75NTR/sortilin signalling was activated in patients with alcohol dependence

**Fig. 1:**
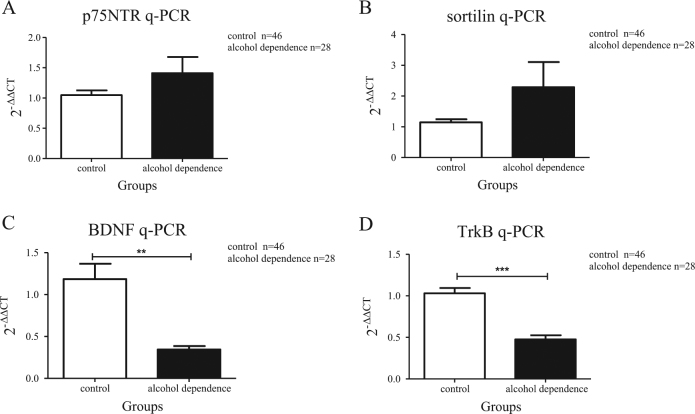
The mRNA levels of *BDNF*, *TrkB*, *p75NTR* and *sortilin* in lymphocytes of patients with alcohol dependence and controls The mRNA levels of *p75NTR* (**a**) and *sortilin* (**b**) were slightly increased; but the mRNA levels of *BDNF* (**c**) and *TrkB* (**d**) were significantly decreased in the lymphocytes of patients with alcohol dependence (*n* = 28) compared to the controls (*n* = 46). Quantitative real-time PCR was performed to measure the mRNA level of each sample, and *β-actin* was measured as control (each measurement was performed in triplicates). All the data were presented in mean ± SEM and analysed by Student’s *t*-test (**P < 0.01, ***P < 0.001)

**Fig. 2:**
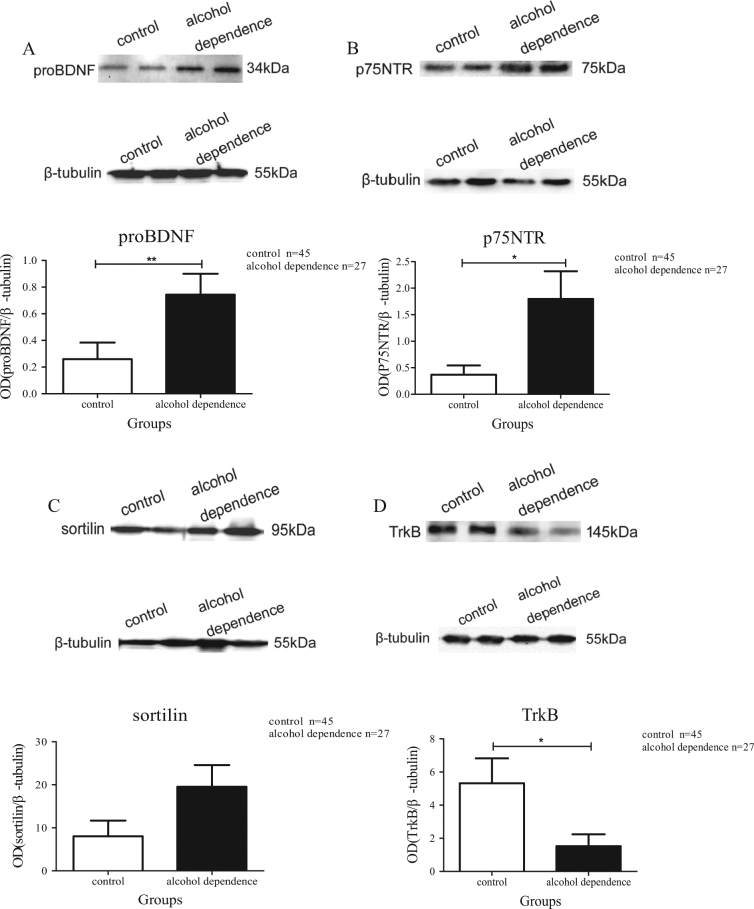
The protein levels of proBDNF, p75NTR, sortilin and TrkB in lymphocytes of patients with alcohol dependence and healthy controls The lymphocytes protein levels of proBDNF (**a**) and p75NTR (**b**) were increased, but not the protein levels of sortilin (**c**). The lymphocytes protein level of TrkB (**d**) was decreased in the alcohol dependence group (*n* = 27) compared to the controls (*n* = 45). Western blots were performed to measure the protein level of each sample, β-tubulin was measured as control. All the data were presented in mean ± SEM and analysed by Student’s *t*-test (*P < 0.05, **P < 0.01)

ProBDNF preferentially binds to its co-receptors (p75NTR and sortilin) to perform the cascade roles in neuronal apoptosis. The lymphocyte mRNA levels of *p75NTR* (*t* = −1.305, *P = *0.227, Student’s *t-*test; Fig. 1a) and *sortilin* (*t* = −1.394, *P = *0.176, Student’s *t-*test; Fig. 1b) were not statistically different from those in the alcohol dependence group (*n* = 28) compared to the control group (*n* = 46). Moreover, the lymphocyte protein levels of proBDNF (*t* = 3.758, *P* = 0.001, Student’s *t-*test; Fig. 2a) and p75NTR (*t* = −2.610, *P* = 0.014, Student’s *t-*test; Fig. 2b) were significantly higher in the alcohol dependence group (*n = *27) than in the control group (*n = *45); while the protein level of sortilin (*t* = 0.706, *P* = 0.494, Student’s *t-*test; Fig. 2c) was not statistically significant between the two groups. The above results suggest that proBDNF, p75NTR and sortilin were activated in patients with alcohol dependence.

### mBDNF/TrkB signalling was deactivated in patients with alcohol dependence

**Fig. 3:**
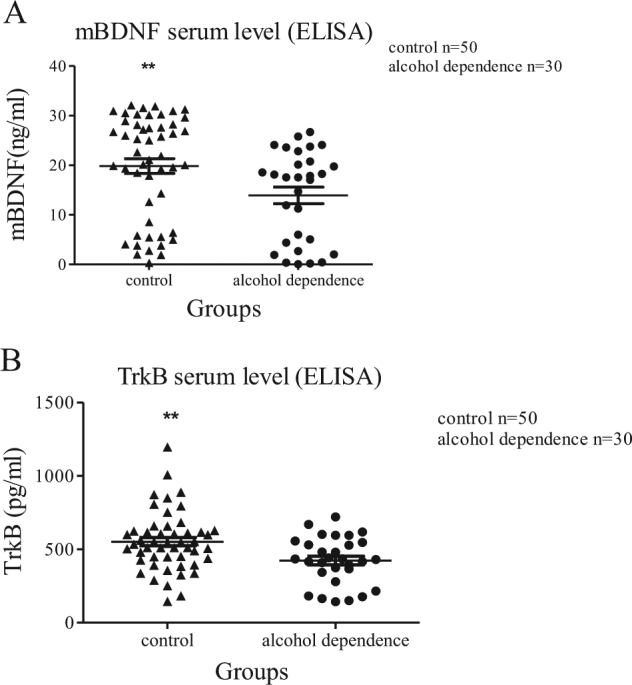
The serum levels of mBDNF and TrkB in patients with alcohol dependence and healthy controls The serum level of mBDNF (**a**) and TrkB (**b**) were decreased in alcohol dependence group (*n* = 30) compared to the controls (*n* = 50). The data of mBDNF were presented in median + IQR and analysed by Mann–Whitney *U*-test. The data of TrkB were presented in mean ± SEM and analysed by Student’s *t*-test (*P < 0.05, **P < 0.01)

By binding to TrkB receptor, mBDNF acts a beneficial role in neuronal survival, which is opposing to proBDNF/p75NTR/sortilin pathway. The lymphocyte mRNA levels of *BDNF* (*t* = −2.804, *P* = 0.01, Student’s *t-*test; Fig. 1c) and *TrkB* (*t* = 5.733, *P* < 0.001, Student’s *t*-test; Fig. 1d) were significantly reduced in the alcohol dependence group (*n* = 28) compared to the control group (*n* = 46). The ELISA result of mBDNF was: *n* = 30, median + IQR = 17.554 + 17.29 ng/ml in alcohol dependence group and the result of health controls was: *n* = 50, median + IQR = 27.334 + 12.55 ng/ml. The serum level of mBDNF was significantly decreased (*z* = −2.982, *P = *0.003, Mann–Whitney *U*-test; Fig. 3a) in alcohol dependence group (*n* = 30) when compared with healthy controls (*n* = 50). The TrkB protein level from lymphocytes was decreased (*t* = −2.598, *P* = 0.013, Student’s *t-*test; Fig. 2d) in the alcohol dependence group (*n* = 27) compared to the control group (*n* = 45). Through the ELISA methods, we also found a reduced TrkB level in alcohol dependence group. The ELISA result of TrkB was: *n* = 30, mean ± SEM = 423.373 ± 29.512 pg/ml in alcohol dependence group and the result of health controls was: *n* = 50, mean ± SEM = 551.873 ± 34.263 pg/ml. The serum level of TrkB was significantly decreased (*t* = −2.984, *P = *0.004, Student’s *t-*test; Fig. 3b) in alcohol dependence group (*n* = 30) when compared with healthy controls (*n* = 50). These results suggested that mBDNF and TrkB are deactivated in patients with alcohol dependence.

### The protein levels of proBDNF/p75NTR/sortilin in lymphocytes were positively correlated with average daily consumption of alcohol

**Fig. 4:**
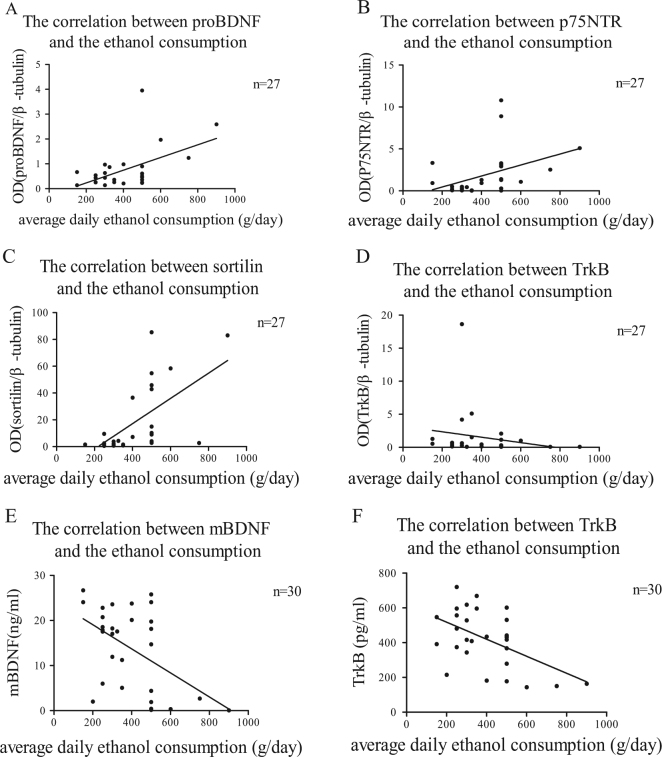
The correlations of average daily alcohol consumption and lymphocyte protein levels of proBDNF, p75NTR, sortilin or TrkB in patients with alcohol dependence The average daily alcohol consumption was positively correlated with the lymphocyte protein levels of proBDNF (**a**, *n* = 27), p75NTR (**b**, *n* = 27) and sortilin (**c**, *n* = 27); but negatively correlated with the lymphocyte protein levels of TrkB (**d**, *n* = 27), serum levels of TrkB (**f**, *n* = 30) and mBDNF (**e**, *n* = 30) in patients with alcohol dependence. All the data were analysed by Spearman’s correlation test

We analysed the relationship between the protein levels of proBDNF/p75NTR/sortilin in lymphocytes and average daily consumption of alcohol. The data showed that the lymphocyte levels of proBDNF, p75NTR and sortilin were positively correlated with the daily ethanol consumption in the alcohol dependence group (*n* = 27; proBDNF: *r* = 0.417, *P = *0.031, Fig. 4a; p75NTR: *r* = 0.476, *P = *0.012; Fig. 4b; sortilin: *r* = 0.708, *P* = 0.001, Fig. 4c; Spearman’s correlation test). These results suggested that chronic excessive alcohol intake might enhance the role of proBDNF/p75NTR/sortilin signalling pathway in alcohol dependence.

### The protein levels of mBDNF/TrkB in lymphocytes and serum were negatively correlated with average daily consumption of alcohol

We further analysed the relationship between the protein levels of mBDNF/TrkB in lymphocytes/serum and average daily consumption of alcohol. The Spearman’s correlation test results showed that the serum level of mBDNF (*n* = 30, *r* = −0.438, *P = *0.015, Fig. 4e) and the lymphocyte/serum level of TrkB (lymphocyte: *n* = 27, r = −0.394, *P* = 0.042, Fig. 4d; serum: *n* = 30, *r* = −0.398, *P = *0.029, Fig. 4f) were negatively correlated with the daily ethanol consumption in the alcohol dependence group. These data suggested that chronic excessive alcohol intake would suppress the mBDNF/TrkB signalling pathway in alcohol dependence.

### The ratio of proBDNF and mBDNF was out of balance in the alcohol dependence patients

**Fig. 5:**
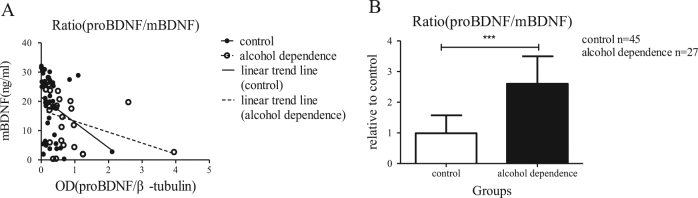
The ratio of proBDNF to mBDNF protein level in the blood of patients with alcohol dependence and of the controls **a** Scattered plot of the protein level of proBDNF in lymphocytes to the protein level of mBDNF in serum in the control group and alcohol dependence group with Spearman’s correlation test. **b** The relative ratio of the protein level of proBDNF in lymphocytes to the protein level of mBDNF in serum was significantly higher in patients with alcohol dependence (*n* = 27) compared to healthy controls (*n* = 45) (Fig. 5b). The data were analysed by Student’s *t*-test (***P < 0.001)

Given that the proBDNF/p75NTR/sortilin pathway and the mBDNF/TrkB pathway acts in an opposing way in the pathogenesis of alcohol dependence, we then analysed the relative ratio of lymphocyte proBDNF (detected by western blot) to the serum mBDNF protein level (detected by ELISA assay) in both alcohol dependence group and control groups. Our results showed that the correlation between proBDNF level and mBDNF level in the control group (*n* = 45, *r* = −0.4238, *P* = 0.0037; Spearman’s correlation test) and in the alcohol dependence group (*n* = 45, *r* = −0.5340, *P* = 0.0041; Spearman’s correlation test) were significant. Furthermore, the relative ratio of proBDNF to mBDNF in the alcohol dependence group (*n* = 27) to the control group was significantly higher than that in the control group (*n* = 45; *t* = 4.135, *P < *0.001, Student’s *t-*test; Fig. 5b), which indicated that the balance of proBDNF and BDNF pathways were dysregulated in patients with chronic ethanol consumption.

## Discussion

Many studies suggest that the reduction of hippocampal volume and cognitive impairment are associated with chronic alcohol consumption in alcohol dependence patients^19,21–24^. The abnormality in BDNF signalling pathway in alcohol dependence may cause the cognitive impairment and brain atrophy. Our ELISA, quantitative real-time PCR and western blotting results proved each other and also supported the hypothesis. We found the results that in alcohol dependence patients, the mRNA levels of *BDNF* and *TrkB* were downregulated which leading to the proteins they translated into changed in line. All the results indicated that the expression of proBDNF/p75NTR pathway was evidently enhanced while mBDNF/TrkB pathway was suppressed in alcohol dependence patients, suggesting that neurotrophic and neurodegenerative balance was broken. ProBDNF binds to p75NTR and sortilin, which plays subsequent biological effects, such as the inhibition of neuronal regeneration and increase in neuronal apoptosis^10,25^, reduction in neuronal migration^11^ and increase in neurite growth collapses^12^. Although in our present experiment, the mRNA and protein levels of sortilin were not elevated significantly in alcohol dependence group due to our small sample size, it does not mean that sortilin is not important in the neurodegeneration roles with proBDNF and p75NTR. The reduced expressions of mBDNF and TrkB would cause reduction of neurogenesis and neuroregeneration. The outcomes of all these neurotrophic dysregulations were likely underlying the cognitive dysfunctions and even brain atrophy in patients with alcohol dependence. As proBDNF and its receptors inhibit the proliferation and migration of neuronal progenitors^11^, the reduction in neurogenesis in alcohol dependence^26,27^ may be likely due to the upregulation of proBDNF and its receptors.

BDNF is not only presented in the nervous system but also in the peripheral blood in humans and rats where the level is higher than that in the brain. Previous studies have shown that BDNF can cross the blood–brain barrier, so the serum level may reflect the level of BDNF in the brain^28,29^, and the BDNF level is relatively stable in the human and other adult primates^30^. However, the results of BDNF expression in serum in the alcohol dependence were not consistent. Some reported no significant changes of BDNF serum level in alcohol dependence patients as compared with healthy controls^31,32^. In contrast, Huang^31^ found a significant increase in serum BDNF. Lee^33^ found that plasma BDNF and NGF levels were higher in patients with alcohol dependence than in the healthy subjects. These conflicting results may be owing to the non-specific detection of both proBDNF and mBDNF with commercial ELISA kits^34^. As proBDNF and mBDNF have different, even opposite functions, it is essential to distinguish mBDNF from proBDNF by ELISA or by western blots. We used our own ELISA kit in this research, which specifically recognised mBDNF rather than the other neurotrophic factors^18,20,34^. With this ELISA kit, we found that the serum mBDNF levels of alcohol dependence patients were lower than the control group. These results suggested that under chronic ethanol exposure, the proBDNF/mBDNF balance was broken down. The enhanced activation of proBDNF/p75NTR signalling pathway may cause neurodegeneration and result in symptoms such as cognitive dysfunction, memory loss and brain atrophy due to the chronic ethanol intoxication. Because mBDNF is a cleaved product from proBDNF, it is reasonable that proBDNF and mBDNF were negatively correlated in both control and alcohol dependence groups. However, the ratio of proBDNF to mBDNF in the alcohol dependence group was much higher than in the control group. This result suggested that the conversion from proBDNF to mature BDNF in the alcohol-dependent subjects was abnormally regulated. Given mBDNF was generated from proBDNF by the cleavage enzyme such as MMP-7 and tPA^35,36^, future studies should elucidate whether proBDNF cleaving enzymes are altered in alcohol dependence.

ProBDNF, mBDNF and their own receptors in the blood of alcoholic subjects can be biomarkers reflecting the amount of ethanol consumption. The ethanol intake was significantly increased in both the CREB heterozygous mice^37^ and BDNF heterozygous rats^38^ in which the BDNF expression was decreased. The degree of brain atrophy was also proportional to alcohol consumption^39^, which also related to the cognitive functions. In the present study, we found that the levels of proBDNF, p75NTR and sortilin in the blood were positively correlated with the average daily alcohol consumption, while the levels of mBDNF and TrkB in the serum were negatively correlated with the daily average alcohol consumption. We speculated that if neurons were severely damaged, and the regeneration was highly inhibited, the mBDNF level would decline, rather than increase. Therefore, proBDNF was directly proportional to the amount of alcohol, indicating that the more alcohol intake, the less the mBDNF was cleaved from proBDNF, and the more severe damage done to the neurons. Therefore, the chronic drinking of patients would have significantly reduced cortical and white matter, because of the increased level of proBDNF, and the reduction of mBDNF. These results suggested that the BDNF related signalling molecules were sensitive biomarkers in response to the alcohol consumption. Specifically, ethanol can enhance the neurodegenerative signals including proBDNF, p75NTR and sortilin, but decrease the neurotrophic signals such as mBDNF and TrkB. Jeanblanc^40,41^ reported that the alcohol intake was increased by K252a (Trk receptor inhibitor) treatment in rats, suggesting that BDNF-mediated signalling cascade might control alcohol intake. This was consistent with our results that the reduction of mBDNF signalling pathway may affect alcohol intake. And it also prompted that the alcohol consumption would impact on the expressions of proBDNF and mBDNF, as well as their cascade receptors.

In summary, the balance between proBDNF/p75NTR/sortilin and mBDNF/TrkB signalling pathways in alcohol dependence was dysregulated. Our findings suggested that the levels of proBDNF, mBDNF and their receptors (p75NTR, sortilin and TrkB) in the blood can be a clue for alcohol dependence research and also therapeutic methods for the future.

Although the present findings are interesting, there are still several limitations in this study: such as single sex (males only), small sample size in each experimental group and patients with only high AUDIT scores. In addition, because our study is based on clinical samples, all the patients recruited receive the oral Oxazepam immediately after hospitalisation. As the blood samples were collected in the next morning 6.00–7.00 a.m. after hospitalization, the effect of the drug and the effect of the alcohol withdrawal (no more than 24 h) on the parameters measured cannot be completely excluded. These limitations may generate considerable errors in data interpretation and conclusion.
